# Oral Health Status and Oral Health Risks Among Tribes in Tamil Nadu, India: An Epidemiological Study

**DOI:** 10.7759/cureus.48721

**Published:** 2023-11-13

**Authors:** Mahalakshmi Kumaraguru, Arthi Balasubramaniam, Meignana Arumugham I

**Affiliations:** 1 Department of Public Health Dentistry, Saveetha Dental College and Hospitals, Saveetha Institute of Medical and Technical Sciences, Saveetha University, Chennai, IND; 2 Department of Public Health Dentistry, Saveetha Dental College and Hospital, Saveetha Institute of Medical and Technical Sciences, Saveetha University, Chennai, IND

**Keywords:** tribals, india, epidemiology, risks, oral health status

## Abstract

Aim

To evaluate the oral health status, self-assessment of oral health, and oral health risks among tribes in Tamil Nadu, India.

Materials and methods

An epidemiological cross-sectional study was carried out among 880 tribals comprising Irulars and Narikuravars. A multistage random sampling method was carried out to select villages from the Vellore district, Tamil Nadu. A World Health Organization (WHO) oral health assessment form, self-oral health assessment form (2013), was administered to collect details regarding the oral health status, self-assessment of oral health, and risks. Descriptive and analytical statistics were performed.

Results

Out of the total study population, 76.4% (n=672) were Irulars and 23.6% (208) were Narikuravars. The decayed, missing, and filled teeth (DMFT) score of the total study population ranged from 0 to 16 and the mean DMFT score of the total population was 5.60 ± 3.416. The mean DMFT score was higher in Irulars (6.58±3.992) than in Narikuravars (5.30±3.160). On assessing the periodontal status, a significant difference (p<0.05) has been observed between the subgroups of age, sex, ethnicity, and education. The prevalence of Leukoplakia and Tobacco Pouch Keratosis in the total study population was 3.3% and 1.6% respectively.

Conclusion

The oral health status of Irulars was poorer than that of Narikuravars. This study highlights the requirement of raising awareness about the significance of oral health and strategically implementing necessary dental care in tribal people living in this region of Tamil Nadu.

## Introduction

Oral health is an operational, structural, aesthetic, biological, and mental state of health and wellness critical to an individual's overall quality of life [1]. Despite the improvement in oral health across the globe, issues remain prevalent among various populations worldwide [2]. There are still inhabitants isolated in natural and pollution-free environments, adhering to their conservative values, traditions, and belief systems such as tribals. Tribes can be defined as indigenous or autochthonous people of the country who function with a common set of social values and principles [3].

Tribals represent a significant indigenous small percentage (9.01%) of the total population constituting India. Approximately 82% of them reside in the central as well as western regions of the nation, with the remaining 11 percent distributed in the southern states [4].

The well-being of any community is affected by the interaction of people's health awareness, cultural, social, demographic, economic, educational, and political factors. Widely accepted belief systems, way of life, myths, customs, and practices related to health, oral health, and disease impact tribal people's health-seeking behavior [5,6].

The tribal people are unsophisticated and backward in terms of pre-agricultural technology, literacy, economy, and education. Their community is stagnant or dwindling in size, and they are isolated from the mainstream of society, living in remote, impenetrable mountainous terrain without infrastructure facilities, and are unchanged by the development process [7]. This underprivileged society, which is scattered across hills, valleys, and plains, has yet to taste the fruits of independence.

According to the 2011 census, there were 794,697 Scheduled Tribes (ST) in Tamil Nadu, with 401,068 males and 393,629 females. In Tamil Nadu, there are 36 ST groups out of which Kattunayakan, Kotas, Irulas, Paniyas, Kurumbas, and Todas are designated as particularly vulnerable tribal groups (PVTG) by the government of India [8].

The word “Irula” is deduced from the Tamil root word “Irul” implying the darkness of night. This could be due to their dark complexion or the fact that all important ceremonies were commonly held at night in the dark [9]. Tiruvallur, Kancheepuram, and Tiruvannamalai districts of Tamil Nadu have approximately 42 percent of the Irular population, followed by Vellore, Viluppuram, and Krishnagiri districts, in which each has roughly 9% percent of the Irular population [8]. Irulas' principal occupations are snake and rat trapping. 

On the other hand, the name Narikuravar is composed of the Tamil words “Nari”-meaning jackal and “Kurava” meaning people. This name relates to their former source of income which was the hunting of wild animals [10]. The government has, however, banned it following the implementation of the Wildlife Protection Act. They now have different jobs and live a nomadic lifestyle, selling beads, toys, and other items in markets, bus stands, and other public places [11]. Most members of this community lack the facility of housing and live on roadsides and in huts.

Traditional healing is always the first choice out of various therapeutic options among these tribes owing to inadequate medical facilities and the easy obtainability of herbal medicines in their local ecosystem [12]. Attributed to differing facilities, human capital, supplies, and geographical distribution, tribal populations use advanced health services at a very low rate [9]. Multiple researches on tribal populations and oral health have been carried out around the world, to name a few examples: the Paniya tribes of Wayanad [13], the Kalpetta tribe [14] in Kerala, the Santal tribals of West Bengal [15]. But even so, the literature on the oral health of the Irula and Narikurava tribes is scarce.

Existing studies on the oral health of Irulars and Narikuravars include those assessing their knowledge, attitude, and practice, healthcare utilization barriers, and individual aspects of oral health such as periodontal status. No study to date has assessed their oral health using a comprehensive and elaborate assessment tool covering all aspects of it. Additionally, no previous study has assessed the self-assessment of oral health and risks among these tribal populations. Organized data on the prevalence of various risk factors is a crucial factor in the execution and planning of oral health prevention and promotion programs that are tailored to the community. By combining clinical data on oral health and risk factors data into a single repository of information, the oral health consequences of socio-behavioral variables can be predicted and appropriate interventional steps can be taken [16]. Ultimately, custom-made oral health programmes meeting the specific needs of the population may be formulated. 

As oral health has a significant impact on general health, this study will contribute to the improvement in the health of these tribal communities. This study aimed to assess the oral health status of Irular and Narikuravar tribes residing in Tamil Nadu, India and the objective was to investigate their self-assessment of oral health and risk.

## Materials and methods

An epidemiological cross-sectional study was conducted among the tribal community in the Vellore district in the month of December 2021. This research was conducted in the villages of Keelpallipattu and Kammasamudram of Vellore district, Tamil Nadu. A multistage random sampling method was adopted for the study. Of 38 districts in Tamil Nadu encompassing a tribal population of 7.94 lakh as per the census 2011 [8], Vellore district was randomly selected by lottery method. From the nine talukas in the Vellore district, Vellore taluk and Vaniyambadi taluk were selected randomly again by lottery method. Keelpallipattu village was selected randomly from Vellore taluk and Kammasamudram village was chosen randomly from Vaniyambadi taluk. From each selected village, all the Irular and Narikuravar households irrespective of their age and gender were included in the study.

All participants were enrolled in the study only after they provided written consent to the study following a thorough explanation of the study’s purpose and procedures involved. People were clustered according to WHO index age groups such as five years, 12 years, 15 years, 35-44 years, and 65-74 years. People who had inhabited the village for more than 365 days were included in the study. All the tribals were enquired orally for medical history and medications. Those who did not give their consent to the study and those whose health was critically compromised at the time of data collection were excluded. Ethical approval was obtained from the scientific review board of Saveetha Dental College, Chennai (IHEC/SDC/PHD-2103/21/079). Official permission to conduct the research in the villages was received from the authorities concerned in each taluk. 

Data collection consisted of two parts. The first part consisted of a questionnaire designed to elicit demographic details of the participants. The second part had the WHO oral health assessment form and self-oral health assessment form (2013) [16] which was administered to gather the data concerning oral health & self-assessment of oral health, and risk. The translated and back-translated questionnaire and proformas were administered in both English and Tamil (vernacular language). For those who were not literate enough to comprehend the questionnaire upon reading, the questions were explained in Tamil. The scores of dentition status have been considered as decay, missing, and filled teeth scores for statistical purposes. The participants were screened for all oral lesions listed in the WHO proforma 2013 [16].

A single pre-trained examiner undertook door-to-door clinical examinations. Assistants were appointed to record the data. To evaluate the intra-examiner reliability, a total of 20 people in a day were subjected to examination. They were re-examined after a minimum of 30 minutes following the first examination. The 20 people selected for re-examination were randomly selected to avoid selection bias. The Cohen's Kappa value obtained for intra-examiner reliability was 0.8 with a good reliability score. After recording the questionnaire, American Dental Association (ADA) type III dental examinations were conducted to record the WHO oral health proforma. The instruments used were sterilized using a standard protocol [16]. 

The recorded data were evaluated using the Statistical Package for the Social Sciences, SPSS Statistics for Windows, Version 23.0 (IBM Corp., Armonk, NY). Descriptive statistics were carried out to obtain the percentages, means, and standard deviations for the data. A Chi-square test, independent t-test, and one-way analysis of variance were done for inferential statistics. P-value ≤0.05 was set as a significant level.

## Results

A total of 880 tribals participated in the study. Table 1 shows the demographic details and oral hygiene aids used by the study subjects. Most of the study subjects (24.5%) were 35-44 years of age and were females (57.3%). The majority of the study population were Irulars (76.4%). About 40% of the study population had completed their primary schooling. No study participants had completed a degree. Most of them (83.6%) use toothbrush and toothpaste to clean their teeth. There was a difference in the distribution of demographic details among the study participants which was statistically significant (p<0.05).

**Table 1:** Demographic details of the study participants

Variable	Sub-variable	N (%)	p-value (Chi-square test)
Age	2-5 years	40(4.5%)	0.000
	6-15 years	120 (13.7%)	
	16-34 years	160(18.2%)	
	35-44 years	216(24.5%)	
	45-64 years	168(19.1%)	
	65-74 years	176 (20%)	
Sex	Male	376(42.7%)	0.000
	Female	504(57.3%)	
Ethnicity	Irular	672(76.4%)	0.000
	Narikuravar	208(23.6%)	
Education	No formal schooling	160(18.2%)	0.000
	Less than primary school	80(9.1%)	
	Primary school	352(40%)	
	Secondary	232(26.4%)	
	Higher Secondary	56(6.4%)	
Oral hygiene aids	Toothbrush and toothpaste	736(83.6%)	0.000
	Finger and brick powder/charcoal/ash	144(16.4%)	

Table 2 demonstrates the subgroup analysis of mean DMFT index scores among the study participants based on age and gender. The DMFT score of the total population ranged from 0 to 16. The mean DMFT of the total population was 5.60 ± 3.416. The mean DMFT score was found to be high among the females in all the age groups. The highest mean DMFT (13.5 ± 4.747) was seen among females of the 65-74 years age group. There was a statistically significant difference in the mean DMFT value distribution among the study participants (p<0.05).

**Table 2:** Sub-group analysis of DMFT scores among the study participants based on age and gender DMFT: Decay, Missing, and Filled Teeth

Age	Males (N)	DMFT Score (Mean ± SD )	Females	DMFT Score (Mean ± SD)	p-value
2-5 years	16	2 ± 0.015	24	6 ± 0.834	0.000
6-15 years	40	6.17 ± 2.929	80	7.04 ± 1.078	
16-34 years	80	5.10 ± 2.483	80	6.80 ± 2.241	
35-44 years	96	6.00 ± 2.691	120	6.27 ± 3.164	
45-64 years	64	5.50 ± 3.805	104	6.54 ± 2.352	
65-74 years	80	11.2 ± 4.126	96	13.5 ± 4.747	

Table 3 shows the comparison of DMFT values between ethnic groups using the independent T-test and between education groups using the One-way ANOVA test. The mean DMFT of the Irular tribe (6.58±3.992 ) was significantly higher (p<0.05) than that of the Narikuravar tribe (5.30±3.160). The mean DMFT was highest among those who had completed up to higher secondary school (7±5.117). A significant difference was seen between the DMFT values of the different education groups.

Table 4 demonstrates the distribution of gingivitis and periodontitis among the study population. The prevalence of gingivitis was higher in the 45-64 years age group (95.2%), females (74.6%), and those with secondary school education (89.7%). Also, the prevalence of periodontitis was found to be high among the 65-74 years age group (76.7%), females (55.6%), and those with no formal schooling (100%). A significant difference (p<0.05) was seen concerning the presence or absence of gingivitis among the different age groups and education groups. With respect to periodontitis, a significant difference (p<0.05) was seen between the subgroups of age, sex, and education. Irulars had a higher prevalence of gingivitis (75%) when compared to Narikuravars (69.2%) though this was not statistically significant (p=0.105). However, a significantly higher prevalence of periodontitis (p=0.003) was noted among Irulars (54.8%) when compared to Narikuravars (42.3%).

**Table 3:** Comparison of mean DMFT values among the study participants based on ethnicity and education DMFT: Decay, Missing, and Filled Teeth

Demographics	Ethnicity	N	DMFT (Mean ± SD)	p-value
Ethnicity	Irular	672	6.58 ± 3.992	0.001
	Narikuravar	208	5.30 ± 3.160	
Education	No formal schooling	160	3.50±2.467	0.000
	Less than primary school	80	5.40±2.514	
	Primary school	352	5.82±3.219	
	Secondary school	232	6.45±3.427	
	Higher secondary school	56	7.00±5.117	

**Table 4:** Distribution of gingivitis and periodontitis among the study population using Chi-square test.

Variable	Sub-variable	Gingivitis N (%)		p-value	Periodontitis N (%)		p-value
		Present	Absent		Present	Absent	
Age	6-15 years	32 (26.6)	88 (73.2)	0.000	0	120 (100)	0.000
	16-34 years	72 (45)	88 (55)		40 (25)	120 (75)	
	35-44 years	176 (81.5)	40 (18.5)		120 (55.6)	96 (44.4)	
	45-64 years	160 (95.2)	8 (4.8)		112 (66.7)	56 (33.3)	
	65-74 years	136 (77.3)	40 (22.7)		135 (76.7)	41 (23.3)	
Sex	Male	272 (72.3)	104 (27.7)	0.487	176 (46.8)	200 (53.2)	0.000
	Female	376 (74.6)	128 (25.4)		280 (55.6)	224 (44.4)	
Ethnicity	Irular	504 (75)	168 (25)	0.105	368 (54.8)	304 (45.3)	0.003
	Narikuravar	144 (69.2)	64 (30.8)		88 (42.3)	120 (57)	
Education	No formal schooling	128 (80)	32 (20)	0.000	160 (100)	0	0.000
	< primary school	80 (100)	0		64 (80)	16 (20)	
	Primary school	272 (77.3)	80 (22.7)		216 (61.4)	136 (38.6)	
	Secondary school	208 (89.7)	24 (10.3)		152 (65.5)	80 (34.5)	
	Higher Secondary	36 (64.2)	20 (35.8)		24 (42.9)	32 (57.1)	

The distribution of leukoplakia and tobacco pouch keratosis among the study population has been tabulated below (Table 5). There was a significant difference seen in the distribution of oral mucosal lesions based on age, sex, and education. Though Irulars had a lower prevalence of Leukoplakia and a higher prevalence of tobacco pouch keratosis when compared to Narikuravars, the difference was not significant.

**Table 5:** Frequency and percentage of leukoplakia and tobacco pouch keratosis among the participants using the Chi-square test

Variable	Sub-variable	N (%)			p-value
		No lesion	Leukoplakia	Tobacco pouch keratosis	
Age	2-5 years	40 (100)	0	0	0.000
	6-15 years	120 (100)	0	0	
	16-34 years	159 (99.4)	1 (0.6)	0	
	35-44 years	207 (95.8)	6 (2.8)	3 (1.4)	
	45-64 years	156 (92.9)	7 (4.2)	5 (3)	
	65-74 years	155 (88.1)	15 (8.5)	6 (3.4)	
Sex	Male	345 (91.8)	24 (6.4)	7 (1.9)	0.000
	Female	492 (97.6)	5 (1)	7 (1.4)	
Ethnicity	Irular	643 (95.7)	18 (2.7)	11 (1.6)	0.181
	Narikuravar	194 (93.3)	11 (5.3)	3 (1.4)	
Education	No formal schooling	144 (90)	2 (1.3)	14 (8.8)	0.000
	< Primary school	78 (97.5)	2 (2.5)	0	
	Primary school	343 (97.4)	9 (2.6)	0	
	Secondary	228 (98.3)	4 (1.7)	0	
	Higher Secondary	44 (78.6)	12 (21.4)	0	

On assessing the distribution of dietary habits among the study population (Table 6), a significant difference (p<0.05) in solid sugar consumption was established between subgroups of age, ethnicity, and education. A significantly (p=0.000) larger percentage of Irulars (67.9%) consumed solid sugars every day when compared to Narikuravars (46.2). Whereas, with regards to liquid sugar consumption, a significant difference(p<0.05) was obtained between subgroups of age, gender, and education. 

**Table 6:** Distribution of dietary habit practices among the study population using the Chi-square test

Variable	Sub-variable	Sugar -N(%)							
		Solid			p-value	Liquid			p-value
		Everyday	Several times a week	Once a week		Everyday	Several times a week	Once a week	
Age	2-5 years	24 (60)	16 (40)	0	0.000	16 (40)	8 (20)	16 (40)	0.000
	6-15 years	56 (46.6)	32 (26.7)	32 (26.7)		80 (66.7)	40 (33.3)	0	
	16-34 years	104 (65)	40 (25)	16 (10)		112 (70)	40 (25)	8 (5)	
	35-44 years	144(66.7)	24 (11.1)	48 (22.2)		128(59.3)	80 (37)	8 (3.7)	
	45-64 years	104(61.9)	48 (28.6)	16 (9.5)		112(66.7)	48 (28.6)	8 (4.8)	
	65-74 years	120(68.2)	24 (13.6)	32 (18.2)		88 (50)	56 (31.8)	32(18.2)	
Sex	Male	224(59.6)	80(21.3)	72(19.1)	0.123	248(66)	112(29.8)	16(4.3)	0.000
	Female	328(65.1)	104(20.6)	72(14.3)		288(57.1)	160(31.7)	56(11.1)	
Ethnicity	Irular	456(67.9)	104(15.5)	112(16.7)	0.000	408(60.7)	208(31)	56(8.3)	0.952
	Narikuravar	96(46.2)	80(38.5)	32(15.4)		128(61.5)	64(30.8)	16(7.7)	
Education	No formal schooling	80(50)	48(30)	32(20)	0.000	96(60)	48(30)	16(10)	0.000
	< primary school	56(70)	16(20)	8(10)		72(90)	8(10)	0	
	Primary school	248(70.5)	56(15.9)	48(13.6)		200(56.8)	136(38.6)	16(4.5)	
	Secondary	152(65.5)	48(20.7)	32(13.8)		128(55.2)	72(31)	32(13.8)	
	Higher secondary	16(28.6)	16(28.6)	24(42.9)		40(71.4)	8(14.3)	8(14.3)	

Table 7 shows the distribution of adverse habits among the study subjects. About 34.5% of Irulars and 26.9% of Narikuravars had the habit of smoking. Also, 50% of Irulars and 50% of Narikuravars had the habit of chewing tobacco. Concerning adverse habit practices, significant (p<0.05) differences were observed between subgroups of education, age, and gender. Most of the males had the habit of smoking and most of the females had the habit of chewing tobacco. A significantly higher percentage (p=0.017) of Irulars (20.2%) reported chewing tobacco every day when compared to Narikuravars (15.4%).

**Table 7:** Distribution of adverse habit practices among the study population

Variable Age	Sub-variable	Adverse Habits- N(%)									
		Smoking				p-value	Chewing tobacco				p-value
		No habit	Everyday	Several times a week	Once a week		No habit	Everyday	Several times a week	Once a week	
	16-34 years	96(60)	24(15)	32(20)	8(5)	0.000	88(55)	24(15)	32(20)	16(10)	0.000
	35-44 years	128(59.3)	64(29.6)	16(7.4)	8(3.7)		56(25.9)	96(44.4)	32(14.8)	32(14.8)	
	45-64 years	112(66.7)	40(23.8)	8(4.8)	8(4.8)		56(33.3)	24(14.3)	56(33.3)	32(19)	
	65-74 years	96 (54.5)	32 (18.8)	40 (22.7)	8(4.5)		80(45.4)	24(13.6)	64(36.4)	8(4.5)	
Sex	Male	88(23.4)	160(42.6)	96(25.5)	32(8.5)	0.000	144(38.3)	9(25.5)	104(27.7)	32(8.5)	0.000
	Female	504(100)	0	0	0		296(58.7)	72(14.3)	80(15.9)	56(11.1)	
Ethnicity	Irular	440(65.5)	128(19)	80(11.9)	24(3.6)	0.161	336(50)	136(20.2)	144(21.4)	56(8.3)	0.017
	Narikuravar	152(73.1)	32(15.4)	16(7.7)	8(3.8)		104(50)	32(15.4)	40(19.2)	32(15.4)	
Education	No formal schooling	160(100)	0	0	0	0.000	160(100)	0	0	0	0.000
	< Primary school	48(60)	32(40)	0	0		40(50)	8(10)	32(40)	0	
	Primary school	192(54.5)	64(18.2)	64(18.2)	32(9.1)		120(34.1)	88(25)	104(29.5)	40(11.4)	
	Secondary	160(69)	40(17.2)	32(13.8)	0		96(41.4)	64(27.6)	32(3.8)	40(17.2)	
	Higher Secondary	32(57.1)	24(42.9)	0	0		24(42.9)	8(14.3)	16(28.6)	8(14.3)	

## Discussion

The beliefs, behaviors, and conventions of individuals have a significant impact on their health [17]. The utilization of health care may be encouraged or discouraged by racial and ethnic ideas and values and research has proved that economically backward groups and ethnic minorities have a lesser propensity towards seeking health services [18].

In our study population, 57.3% were females and 42.7% were males. Similar results were observed among Telangana tribes, where female participation (57%) was higher than male participation [6]. Regrettably, 40% of the study population had completed only up to primary school and only 6.4% had completed higher secondary school. Likewise, in a study conducted on Narikuravars of Pondicherry, 91% of the population was uneducated and only 8.3% had attended primary school [11]. This may be due to their nomadic lifestyle, where parents have turned to various jobs like selling beads, toys, and other items in markets, bus stands, fairs, and other places where their kids would never get the chance to attend school [10]. Children are introduced to earning activities at a very young age, and people are unmotivated to send their children to school. Contrary to our expectations, 83.6% of the study population used toothbrush and toothpaste. It may have been preferable for participants to respond to questions about dental health and behave in a socially acceptable manner, among other things, which may have influenced the participants' responses. However, more than half (55.47%) of the Paniya tribals used a brush to clean their teeth [10]. In contrast, 46.4% of Lambada tribes, used toothbrushes, 30% used twigs and only 9.4% used fingers to clean their teeth [6].

Dental caries are mostly seen as a result of prosperity and civilization. Geographically remote and primitive populations put themselves at significant risk because of their excessive susceptibility to dental caries and insufficient exposure to modernity. Passive oral health care, inadequate usage, a lack of awareness, poverty, and illiteracy are a few of the causes of the same.

In our study, the mean DMFT of females in the 65-84-year age group was 13.5 ± 4.747. This is in accordance with a study conducted on Odisha tribes [19] where the mean DMFT was highest in the 65-84-year age group (7.56 ± 4.29) and least in the 2-5-year-old group (0.0). Also, a study conducted on Paniya tribes of Kerala [10] reported the mean DMFT of the 65-84-year group was much higher (18.47 ± 13.10) and the mean DMFT of the 35-44-year group was much lower (1.52 ± 1.95) when compared to the results of our current study. 

Secondly, contrary to our expectations, mean DMFT was highest (7.00 ± 5.12) in those who had completed up to higher secondary school rather than the lower-level education groups. This could be ascribed to higher income jobs in those with higher literacy, ultimately exposing them to refined, processed foods, leading to the development of caries. However, further longitudinal studies assessing a multitude of other possible covariates with respect to the link between DMFT and education are required to establish a strong conclusion. Another possible reason could be the decreased awareness among school children owing to a lack of oral health education and promotion, ultimately resulting in poor oral health drive. Moreover, in the present study, the Irula tribes had a higher mean DMFT (6.58 ± 3.99) than the Narikuvar tribe (5.30 ± 3.160). This is not surprising given that Irulars, one of the particularly vulnerable tribal groups (PVTGs) defined by the Indian government, are known for their enduring backwardness, which is shown in their subpar standard of life, eating habits, health, and hygiene [20]. 

In the present study, subgroups with the highest percentage of gingivitis were those belonging to the 45-64 age group (95.2%, n=160), Irular tribe (75%, n=504), less than primary school group (100%, n=80) and females (74.6%, n=376). However, in a study conducted by Kumar et al. [19] individuals exhibiting bleeding gums were majorly present in the age group of 35-44 years. The highest frequency of periodontitis was seen in the 65-84-year age subgroup (n=135, 76.7%), females(n=280, 55.6%), Irular tribe (n=368, 54,.8%), and in the no formal schooling subgroup (n=160,100%). However, in a study conducted on tribals of Odisha [19], shallow pockets were predominant in those who were 35-44 years old whereas those belonging to the 65-84 years age group exhibited deep pockets. In our study, the periodontal status deteriorated with age, which also holds for the Bhil tribes of Rajasthan [4].

Moreover, 76.9% of adults in the Paniya tribe had periodontal disease [9] which is much higher compared to the prevalence reported in the current study among Irulas (54.8%) and Narikuravars (42.3%). This could be attributed to the high percentage of tribals who smoked in the Paniya tribe in comparison with the Irulars and Narikuravars. In this study, the prevalence of leukoplakia was 3.3% and that of Tobacco Pouch Keratosis was 1.6%. This is much higher than the prevalence of leukoplakia among the Paniya tribes of Kerala which was 1.90% [9]. Similarly, in contrast to our study, the prevalence of Tobacco Pouch keratosis (4.8%) was much higher among Juang tribes of Odisha [20].

In this study, the solid and liquid sugar consumption frequency was significantly different between subgroups of age, sex, and education. In a study conducted on Oraon tribes of Jharkhand [21], those who snacked in between two meals ate a variety of prepared items, both homemade and bought from stores such as papad (55.6%), biscuits (61.1%), tea (80%), puffed rice (30.6%), fruit (30.6%), rice flakes (22.2%), sattu ka pani (roasted gram powder mixed with water; 2%), and others (5.6%). The simple accessibility of sugar-sweetened drinks (SSDs) in both urban and rural settings dramatically raises per capita consumption [22]. However further studies considering indigenous food habits and sugar consumption patterns are mandated to establish causal relationships.

In the present study, 54.5% of individuals in the 65-84 years age group chewed tobacco. These results align with a study conducted exclusively on elderly tribals aged 60 and above in Kalpetta, where 69.3% used chewing tobacco [23,24]. In a study conducted by Valsan et al. [10], the Paniya population reported excessive use (89.3%) of paan masala. Among Santhal tribals of West Bengal, various products consisting of smokeless tobacco were consumed by 41.7% of the population, and 9.7% had the habit of both smoking and consuming smokeless tobacco [23].

The present study clearly explains the oral health burden among the tribes in the Vellore district of Tamil Nadu. The data has brought to light that the implementation of a basic oral health program for the population is of utmost importance. The program should cover free-of-cost emergency and basic preventive dental treatment at affordable rates which could be provided by adequately educated tribal primary oral health care personnel. This can aid in bridging the gap between tribal people and their motivation to seek dental services. Such a well-designed program must reach the depths of tribal settlements with active participation from local men trained in medicine and tribal heads. Finally, yet most importantly, poor oral health can be attributed largely to cultural and traditional customs and these programs direct their focus towards the transformation of unhealthy and backward customs while retaining the healthy ones.

Limitations

This study produced only limited evidence owing to its cross-sectional nature. Further longitudinal studies assessing long-term trends are mandated. The availability and accessibility of oral health care services in the villages which could have had a significant link with the high prevalence of oral diseases were not explored as potential covariates in the current study.

## Conclusions

In this study, Irulars had a significantly higher mean DMFT and higher prevalence of periodontitis, indicative of poorer oral health status of Irulars in comparison with that of Narikuravars. Moreover, compared to Narikuravars, a significantly higher proportion of Irulars consumed solid sugars and chewed tobacco daily, bringing to light their lack of awareness of its oral health effects. This study has yielded valuable results that could serve as a foundation upon which health authorities and dental professionals can plan strategies for oral health promotion, prevention, and treatment among the Irular and Narikuravar populations by providing preventive dental aids at affordable cost. Special focus must be provided on educating Irulars on harmful eating practices and the dire consequences of adverse habits. Oral health programs must aim to reduce the high prevalence of caries and periodontitis in these populations. Further, longitudinal studies are mandated to identify the reason for the difference in oral health status between the two groups as well as to explore other factors that might play a crucial role in their oral health.

## References

[1] Viragi PS, Dwijendra KS, Kathariya MD, Chopra K, Dadpe MV, Madhukar HS (2013). Dental health and treatment needs among children in a tribal community. J Contemp Dent Pract.

[2] Yadav P, Kaur B, Srivastava R, Srivastava S (2014). Oral health disparities: review. IOSR J Dent Med Sci.

[3] Butani Y, Weintraub JA, Barker JC (2008). Oral health-related cultural beliefs for four racial/ethnic groups: assessment of the literature. BMC Oral Health.

[4] Kumar TS, Dagli RJ, Mathur A, Jain M, Balasubramanyam G, Prabu D, Kulkarni S (2009). Oral health status and practices of dentate Bhil adult tribes of southern Rajasthan, India. Int Dent J.

[5] (2023). Religion in India: tolerance and segregation. (2021). Accessed: January. https://www.pewresearch.org/religion/2021/06/29/religion-in-india-tolerance-and-segregation/..

[6] Asif SM, Naheeda S, Assiri KI (2019). Oral hygiene practice and periodontal status among two tribal population of Telangana state, India- an epidemiological study. BMC Oral Health.

[7] Bhadradri Kothagudem is all (2023). Bhadradri Kothagudem Is All About ‘Inclusive Development. https://niti.gov.in/index.php/bhadradri-kothagudem-all-about-inclusive-development.

[8] (2023). Census of India 2011, national population register & socio-economic and caste census. (2011). Accessed. https://censusindia.gov.in/nada/index.php/catalog/42619..

[9] Sathiyanarayanan S, Muthunarayanan L, Devaparthasarathy TA (2019). Changing perspectives in tribal health: rising prevalence of lifestyle diseases among tribal population in India. Indian J Community Med.

[10] Valsan I, Joseph J, Janakiram C, Mohamed S (2016). Oral health status and treatment needs of Paniya tribes in Kerala. J Clin Diagn Res.

[11] Muthanandam S, Babu BV, Muthu J, Suganya R, Vezhavendhan N, Kishore M (2021). Assessment of knowledge, awareness, and attitude towards oral precancer and cancer among Narikuravar population in Pondicherry state. South Asian J Cancer.

[12] Kumar MM, Pathak VK, Ruikar M (2020). Tribal population in India: a public health challenge and road to future. J Family Med Prim Care.

[13] Deepan Kumar CV, Mohamed S, Janakiram C, Joseph J (2015). Validation of dental impact on daily living questionnaire among tribal population of India. Contemp Clin Dent.

[14] Janakiram C, Joseph J, Vasudevan S (2016). Prevalence and dependency of tobacco use in an indigenous population of Kerala, India. J Oral Hyg Health.

[15] Mandal S, Ghosh C, Sarkar S, Pal J, Kar S, Bazmi BA (2015). Assessment of oral health status of Santal (tribal) children of West Bengal. J Indian Soc Pedod Prev Dent.

[16] Petersen PE, Baez RJ (2013). Oral health surveys: basic methods, 5th ed. https://iris.who.int/handle/10665/97035.

[17] Tripathi N, Kishore J, Babu B (2020). Prevalence of hypertension in Indian tribal adult population: a scoping review. J Adv Res Med.

[18] Rodriguez-Galan MB, Falcón LM (2009). Perceived problems with access to medical care and depression among older Puerto Ricans, Dominicans, other Hispanics, and a comparison group of non-Hispanic Whites. J Aging Health.

[19] Kumar G, Rai S, Jalaluddin M, Tripathi RM, Bagchi A, Tiwari R (2021). Assessment of oral health status and treatment needs amongst the tribals residing in Northern Bhubaneswar, Odisha. J Family Med Prim Care.

[20] Das D, Suresan V, Jnaneswar A, Khurana C, Bhadauria US, Saha D (2019). Oral health status and treatment needs among the Juang tribe-a particularly vulnerable tribal group residing in Northern Odisha, India: a cross-sectional study. Health Soc Care Community.

[21] Ghosh-Jerath S, Singh A, Lyngdoh T, Magsumbol MS, Kamboj P, Goldberg G (2018). Estimates of indigenous food consumption and their contribution to nutrient intake in Oraon tribal women of Jharkhand, India. Food Nutr Bull.

[22] Gulati S, Misra A (2014). Sugar intake, obesity, and diabetes in India. Nutrients.

[23] Haque HZ, Pal D, Sadhukhan SK, Das S (2021). A cross-sectional study on oral hygiene among Santhal tribal adults in a rural area of West Bengal. J Family Med Prim Care.

[24] Neelamana SK, Janakiram C, Varma B (2020). Oral health status and related quality of life among elderly tribes in India. J Family Med Prim Care.

